# Individual and Cooperative Photochemical–Enzymatic Processes for the Degradation of the Dye Bromothymol Blue: Kinetic and Eco-Toxicity Analysis

**DOI:** 10.3390/ijms27177767

**Published:** 2026-08-30

**Authors:** Andrea S. Urquiza, Agustina Reynoso, M. Alicia Biasutti, Hernán A. Montejano, Eugenia Reynoso

**Affiliations:** 1 Departamento de Química, Facultad de Ciencias Exactas, Físico-Químicas y Naturales, Universidad Nacional de Río Cuarto (UNRC), Ruta Nacional 36 Km 601, Río Cuarto X5804BYA, Córdoba, Argentina; aurquiza@exa.unrc.edu.ar (A.S.U.); areynoso@exa.unrc.edu.ar (A.R.); abiasutti@exa.unrc.edu.ar (M.A.B.); 2 Instituto de Investigaciones en Tecnologías Energéticas y Materiales Avanzados (IITEMA), CONICET–UNRC, Ruta Nacional 36 Km 601, Río Cuarto X5804BYA, Córdoba, Argentina; 3 Instituto para el Desarrollo Agroindustrial y de la Salud (IDAS), CONICET–UNRC, Ruta Nacional 36 Km 601, Río Cuarto X5804BYA, Córdoba, Argentina

**Keywords:** sulfone phthalein dye, UVC photolysis, laccase-mediated oxidation, sequential (hybrid) treatment, bioassay

## Abstract

The degradation of bromothymol blue (BTB) in aqueous solution was investigated through individual and sequential photochemical and enzymatic treatments. Photodegradation experiments were performed under UVC, UVB, UVA and visible irradiation at different pH values and atmosphere conditions, while enzymatic degradation was evaluated using Laccase from *Trametes versicolor* under varying pH, temperature and enzyme concentration. Kinetic analyses were performed in all cases. BTB degradation was strongly dependent on irradiation wavelength and pH. The highest photodegradation rates were obtained under UVC irradiation, particularly in alkaline medium. Additionally, our results suggest that BTB photolysis mainly proceeds through a unimolecular pathway. On the other hand, enzymatic degradation was favored at acidic pH, elevated temperature, and high amount of laccase, with optimal performance observed at pH 5 and 40 °C. Sequential treatments combining photochemical and enzymatic processes improved the overall removal efficiency, reaching degradation values above 70% regardless of the treatments order. Ecotoxicological evaluation using the *Vibrio fischeri* bioluminescence inhibition assay revealed a significant reduction in toxicity after all treatments, particularly those involving UVC irradiation. These results demonstrate the complementary nature of both processes and highlight the potential of combined photochemical–enzymatic treatments for the remediation of dye-contaminated waters.

## 1. Introduction

Water contamination by industrial effluents constitutes one of the most pressing environmental challenges worldwide, particularly due to the continuous discharge of recalcitrant organic pollutants. Among these, synthetic dyes are of particular concern due to their extensive use and high production rates, exceeding 7 × 10^5^ tons annually and encompassing more than 10,000 different compounds [1,2]. It is estimated that 10–50% of dyes are released into wastewater during manufacturing and application processes, especially in the textile industry, generating nearly 200 billion liters of colored wastewater annually, of which up to 50% may be discharged directly into aquatic ecosystems without adequate treatment [3,4]. The presence of dyes in aquatic environments can impair light penetration and photosynthetic activity even at low concentrations (<1 mg L^−1^), while many dyes and their transformation products may exhibit toxic, mutagenic, or carcinogenic effects and persist due to their complex aromatic structures and resistance to conventional treatment processes [1,2,3,4].

Consequently, physical, chemical, and biological technologies have been investigated for dye removal. Physical processes such as adsorption, membrane filtration, and ion exchange can achieve high removal efficiencies but are generally non-destructive and generate secondary waste streams, whereas chemical treatments, including coagulation-flocculation, electrochemical processes, photochemical treatments, and advanced oxidation processes, can promote degradation but may involve high energy or operational costs and the formation of potentially toxic byproducts [5,6]. In contrast, biological approaches have attracted increasing attention due to their low environmental impact, reduced sludge generation, and operation under mild conditions, with enzymatic systems offering additional advantages such as high catalytic efficiency and substrate specificity [7,8,9].

Among enzymatic alternatives, oxidoreductases such as laccases have emerged as promising biocatalysts for dye degradation [8,9]. Laccases are multicopper oxidases capable of oxidizing a broad range of phenolic and non-phenolic substrates using molecular oxygen as the final electron acceptor, producing water as the main byproduct [3,4,8,9]. These enzymes can generate reactive radical intermediates, which may undergo further transformation reactions, enhancing pollutant degradation [4,9]. However, their application is often limited by enzyme instability under harsh operational conditions and requires strategies to improve stability and reusability [7,10].

In this context, combined treatment systems have emerged as a promising approach to overcome the limitations of individual methods. These systems integrate two or more processes, enhancing degradation efficiency, improving biodegradability, and reducing overall costs [2,7]. Namely, if the transformation products generated during one process exhibit high reactivity toward the subsequent process, the combination of both degradation methods may substantially enhance contaminant removal and modify the distribution of the resulting products. Therefore, the investigation of the combined photolytic and enzyme-catalyzed degradation process of organic contaminants is of great importance.

Based on these considerations, and under the premise that the efficiency of individual treatments can be enhanced through their strategic combination, the present work aims to investigate the degradation of bromothymol blue (BTB) in aqueous media by photochemical and enzymatic processes, as well as their sequential combination.

BTB is a sulfone phthalein dye that exhibits distinct pH-dependent color transitions, appearing yellow in acidic media and blue in alkaline conditions, with a green coloration near neutral pH due to the coexistence of different ionic species (pK_a_ ~ 7.5) [11,12,13]. This well-defined acid–base and spectral behavior facilitates the monitoring of its transformation under different experimental conditions and provides a useful framework for studies of photochemical and enzymatic processes. Despite its widespread laboratory and industrial use, exposure to BTB has been associated with adverse health effects, including irritation of the respiratory system, skin, and eyes, as well as potential damage to internal organs upon prolonged exposure [14]. Previous studies have explored BTB decontamination through adsorption, oxidation, and photocatalysis, reporting significant removal efficiencies under optimized conditions [14,15,16]. However, to the best of our knowledge, the sequential combination of photochemical and enzymatic treatments has not yet been investigated for BTB. Furthermore, most previous studies have primarily focused on BTB removal efficiency, whereas the potential toxicity of the resulting transformation products has received considerably less attention. This distinction is particularly relevant because contaminant degradation does not necessarily imply a reduction in ecological risk.

Accordingly, the present work investigates BTB degradation through UV irradiation and laccase-catalyzed oxidation, as well as their sequential combination, considering the influence of key operational parameters including pH, light source, oxygen availability, temperature, and enzyme concentration. Furthermore, two sequential configurations were evaluated, consisting of photochemical treatment on the products of enzymatic degradation, and enzymatic treatment on the products of photochemical degradation, using the optimal conditions established for each individual process.

The treated solutions were further subjected to ecotoxicological assessment using a bioluminescence inhibition assay, allowing degradation efficiency to be evaluated together with the potential toxicity of the resulting transformation products.

Overall, this approach provides an integrated evaluation of individual and combined treatments and contributes to the identification of efficient and environmentally safer strategies for BTB removal, while providing a basis for exploring combined treatment approaches for structurally related dyes.

## 2. Results and Discussion

### 2.1. Photochemical Treatment

**Figure 1:**
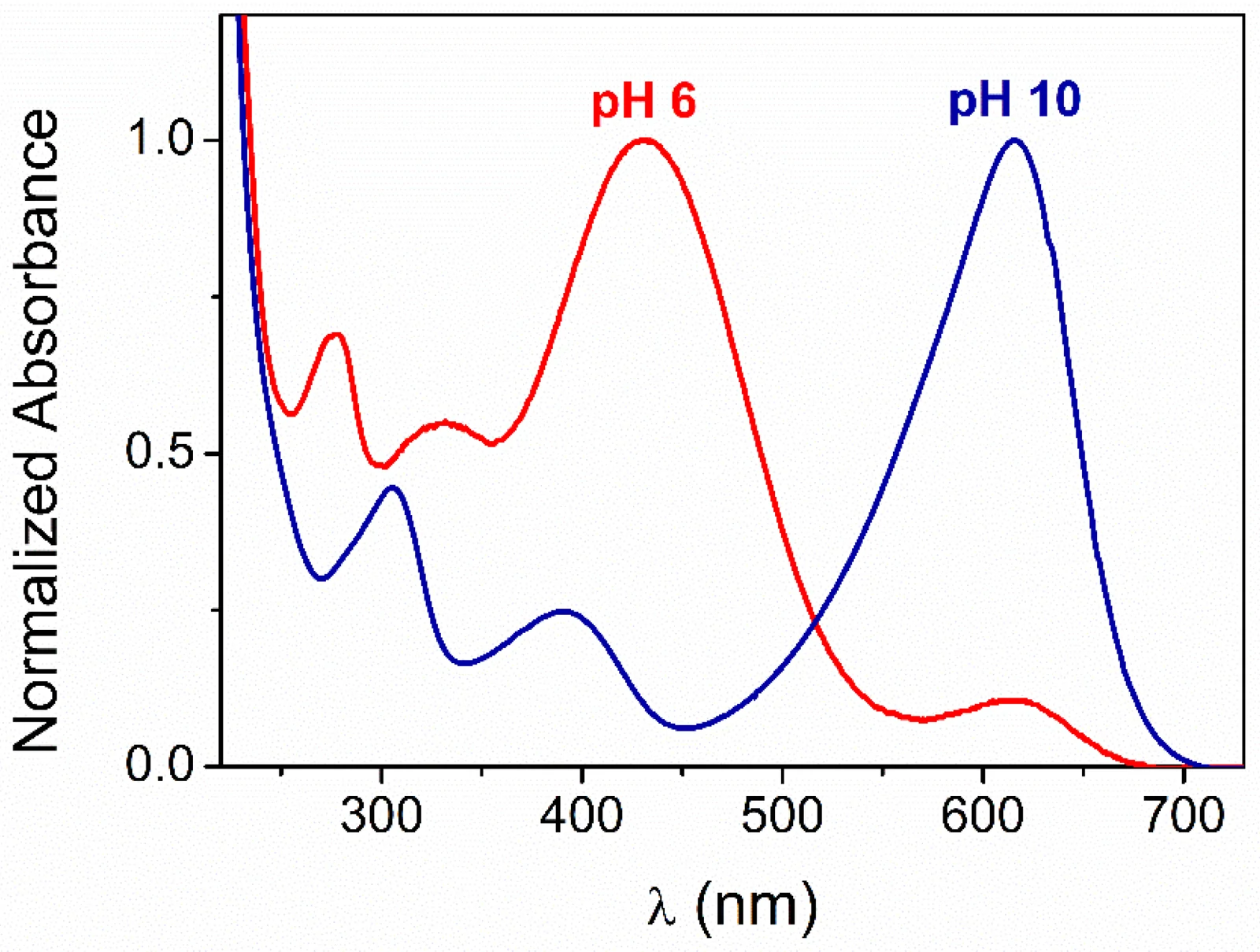
Normalized absorption spectra of BTB in buffer solutions of pH 6 and pH 10.

Figure 1 displays the normalized UV–Visible absorption spectra of BTB in buffered solutions at pH 6 and 10. These pH values were selected because they are sufficiently distant from the pK_a_ (~7.5) [11,12,13], ensuring that the protonated and deprotonated forms of BTB predominate at each condition. In both media, the dye exhibits a broad absorption band in the UV–Visible region; however, the spectral profiles differ markedly, indicating the predominance of different acid–base species at each pH.

At pH 6, the monovalent anionic form predominates, showing an absorption maximum centered at 432 nm. In contrast, at pH 10, the dominant species corresponds to the dianion, whose extended conjugation relative to the monoanionic form results in a bathochromic shift, with a principal absorption band at 615 nm [12,13].

**Scheme 1:**
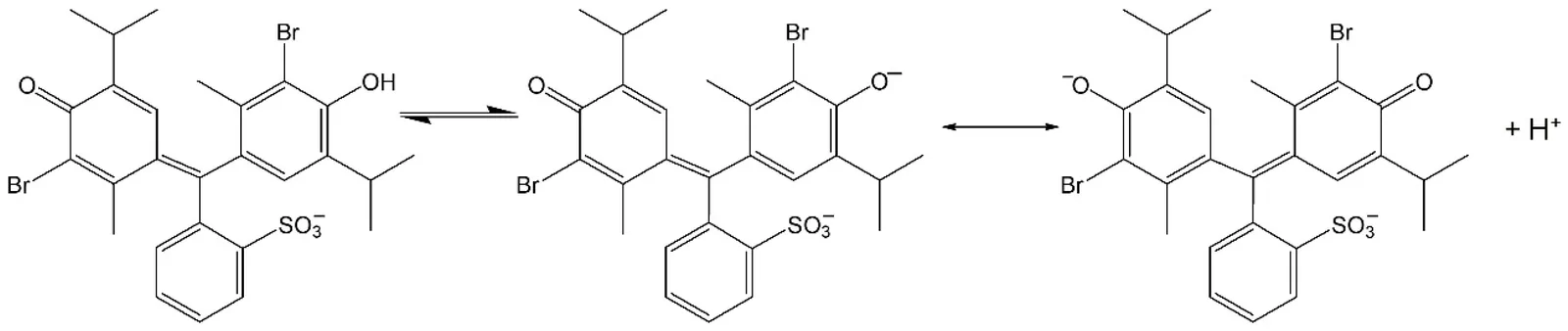
Deprotonation equilibrium of bromothymol blue (BTB); pK_a_ ~ 7.5 [12].

Scheme 1 illustrates the deprotonation equilibrium of BTB, including the resonance-stabilized structure in alkaline medium.

To investigate the influence of irradiation wavelength, pH, and dissolved oxygen on the photodegradation process, BTB solutions (25 μg mL^−1^) were irradiated with UVC, UVB, UVA, and Visible light at pH 6 and 10 under both air and argon atmospheres. The evolution of the absorption spectra was monitored as a function of irradiation time at each experimental condition.

Kinetic analysis was performed by monitoring the decrease in absorbance at 432 nm (pH 6) and 615 nm (pH 10), corresponding to the maxima of the predominant species. The absorbance data were fitted according to Equation (1).

**Figure 2:**
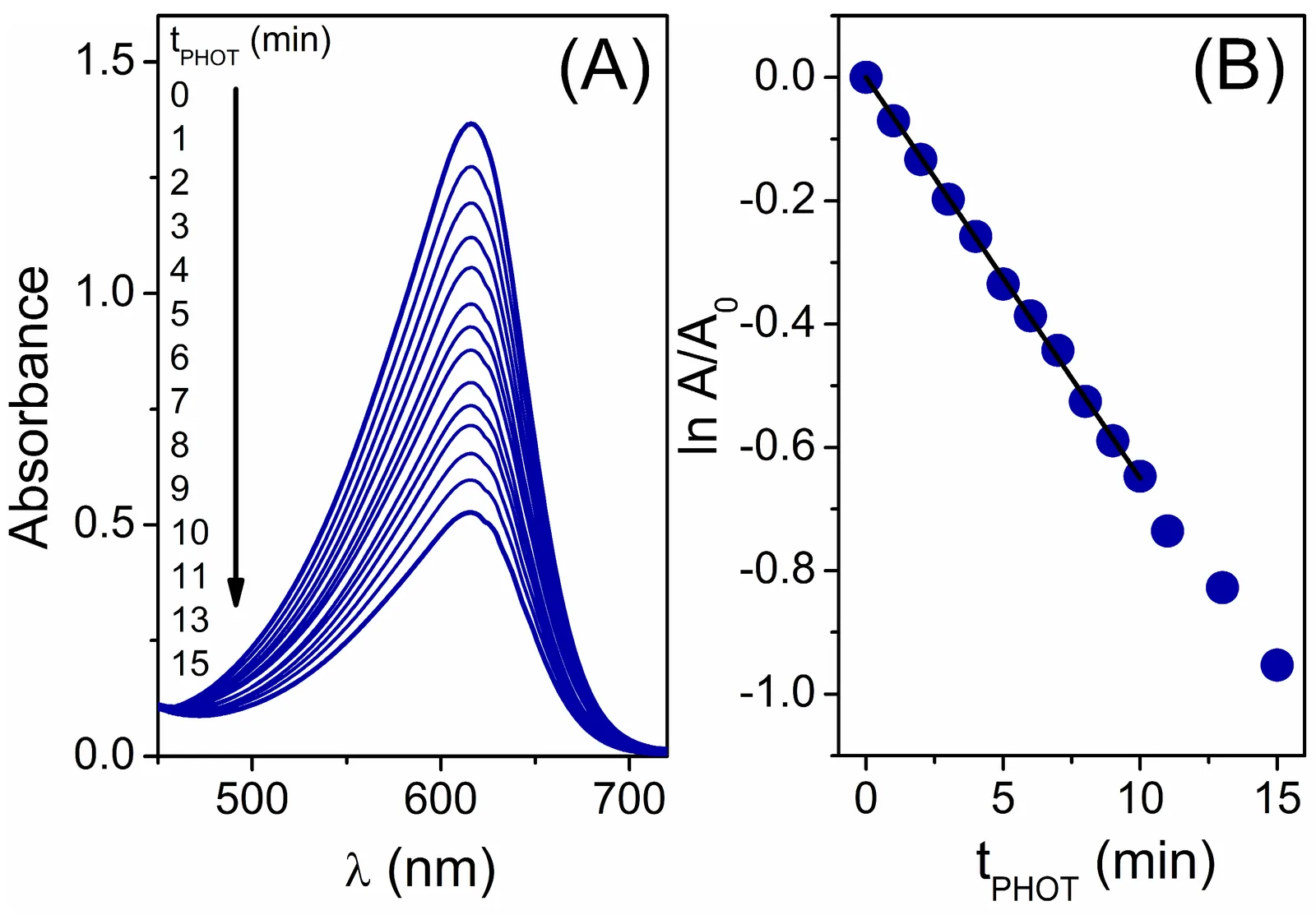
(**A**) Evolution of the absorption spectrum of BTB (25 μg mL^−1^) at pH 10 under UVC irradiation in argon atmosphere. (**B**) Graphic representation of Equation (1) for the determination of the apparent kinetic photodegradation constant, *k*_PHOT_; the solid line represents the linear regression of the experimental data.

Representative results are presented in Figure 2. Figure 2A shows the spectral evolution of BTB at pH 10 under UVC irradiation in an argon atmosphere. A progressive decrease in absorbance is observed, indicating photodegradation of the dye. The corresponding linear plot of ln (*A*/*A*_0_) versus time, which allows the determination of the apparent photodegradation rate constant *k*_PHOT_, is shown in Figure 2B. 

**Figure 3:**
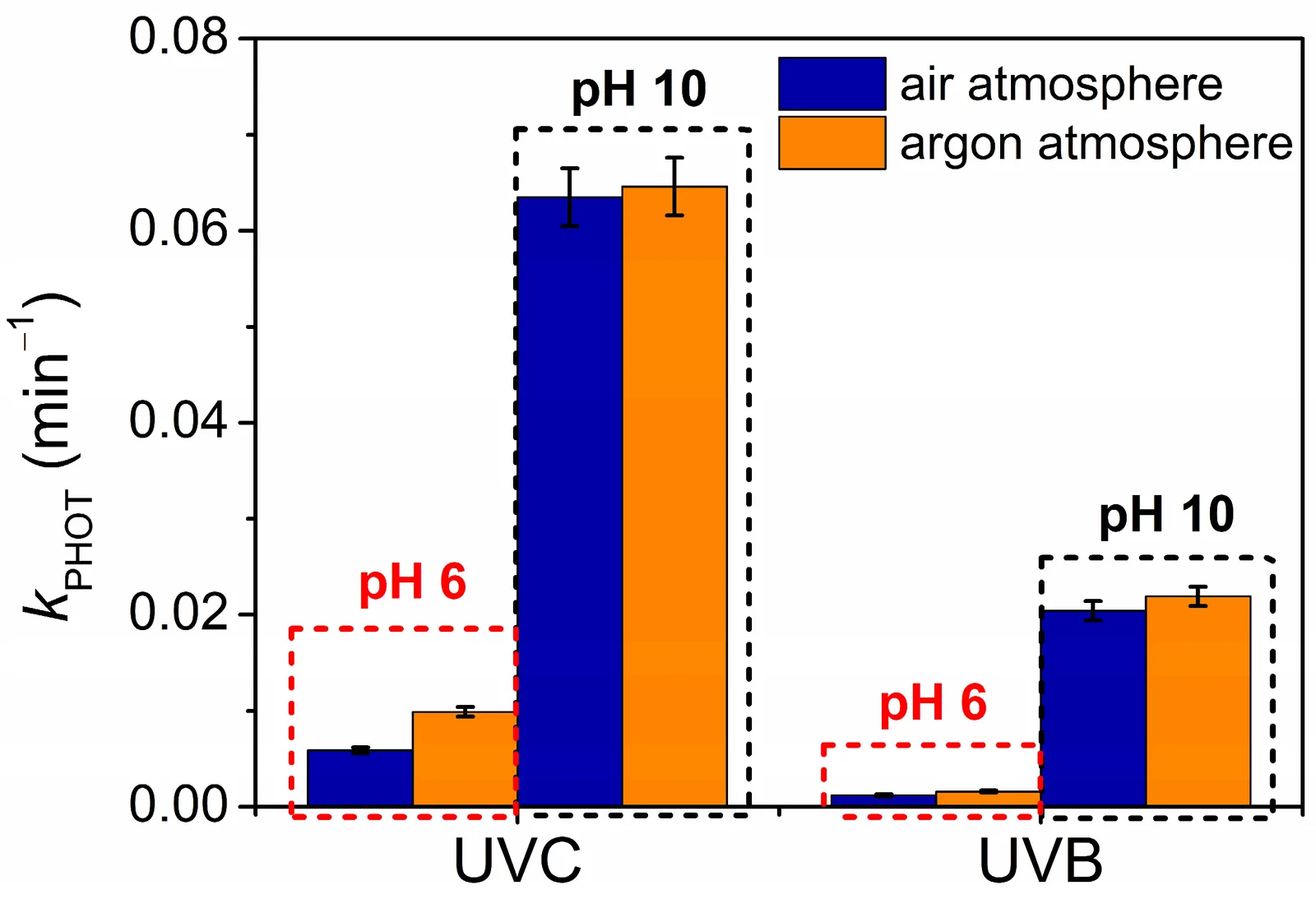
Apparent photodegradation constants of BTB (*k*_PHOT_) obtained by irradiation with UVC and UVB light at pH 6 and pH 10 and in air- and argon-saturated solutions. Error bars represent ±1 standard deviation (SD) calculated from triplicate measurements.

For comparative purposes, the *k*_PHOT_ values obtained under different irradiation wavelengths, pH conditions, and atmospheres are summarized in Figure 3 and Table S1 (Supplementary Materials). No significant spectral changes were detected under UVA or visible irradiation at any of the pH values or atmospheric conditions investigated; therefore, *k*_PHOT_ values could not be reliably determined and are not included in the comparison.

The kinetic results demonstrate that BTB is susceptible to degradation under UVC and UVB irradiation at both pH values. However, significant differences emerge when comparing species and experimental conditions. The dianionic form (pH 10) exhibits consistently higher *k*_PHOT_ values than the monoanionic species (pH 6), indicating greater photoreactivity under alkaline conditions. Additionally, UVC irradiation produces the highest degradation rates, highlighting the decisive role of photon energy in promoting the process. This behavior can be rationalized by the emission characteristics of the lamps used: the UVC source emits predominantly at 254 nm, whereas the UVB lamp (nominally 300 nm) also exhibits a significant contribution in the UVA region (around 364 nm), resulting in a mixed UVB-UVA output with lower effective photon energy.

The absence of measurable degradation under UVA and visible light suggests that BTB is highly photostable under lower-energy irradiation, irrespective of pH or oxygen availability. This behavior is consistent with the intrinsic photostability of many synthetic dyes, which are structurally designed to resist light-induced discoloration.

Finally, a comparison between air-saturated and argon-purged solutions provides insight into the possible contribution of oxygen to the degradation process. At pH 10, no significant differences in *k*_PHOT_ are observed between the two atmospheres under UVC or UVB irradiation. Thus, under the conditions investigated, molecular oxygen does not appear to play a rate-determining role in the degradation of the dianionic form. This observation is consistent with a predominantly unimolecular photodegradation pathway, although it does not by itself exclude a concurrent contribution from oxygen-mediated processes.

Conversely, at pH 6, a modest but reproducible decrease in *k*_PHOT_ is observed in air-saturated solutions compared with argon-purged solutions, suggesting that dissolved oxygen may partially influence the photodegradation of monoanionic species. This behavior is consistent with the coexistence of parallel degradation routes. Based on these observations, one possible interpretation is that molecular oxygen interacts with the excited state of BTB, reducing the efficiency of the predominant unimolecular photodegradation pathway.

To further investigate this aspect, and considering that reactive oxygen species (ROS) may be generated under irradiation and that indirect photolysis may therefore contribute to BTB degradation, the *k*_PHOT_ values were determined under air atmosphere in the absence and in the presence of the ROS scavenger’s isopropyl alcohol (IPA), sodium azide (NaN_3_), superoxide dismutase (SOD), and catalase (CAT). The corresponding pseudo-first-order kinetic plots are shown in Figure S1 (Supplementary Materials). These experiments were performed exclusively at pH 6 because, under this condition, differences in *k*_PHOT_ were observed between air- and argon-saturated atmospheres. UVB irradiation was used instead of UVC to minimize potential interference associated with the absorption of radiation by the inhibitors employed in this study.

Figure S1 clearly shows that the presence of IPA, NaN_3_, and CAT decreased the BTB degradation rate, whereas no significant change was observed in the presence of SOD. These results suggest that hydroxyl radical (HO^•^), singlet oxygen (O_2_(^1^Δ_g_)), and hydrogen peroxide (H_2_O_2_) contribute to BTB photodegradation through a self-sensitized process.

Although these experiments demonstrate the generation and involvement of ROS in BTB photolysis, their contribution appears insufficient to significantly influence the overall degradation process under the experimental conditions investigated. Thus, direct and indirect photolytic pathways likely coexist, but the ROS-mediated pathway does not appear to kinetically compete with direct photolysis. However, this interpretation should be considered a mechanistic hypothesis rather than a demonstrated reaction process.

In a comprehensive review, Anisuzzaman et al. compiled extensive data on the photochemical treatment of dyes belonging to different chemical families, many of which are widely employed in the textile industry [17]. The review indicates that significant degradation by direct photolysis is generally achieved only under high-energy irradiation, particularly UVC. Furthermore, the efficiency of direct photolysis strongly depends on operational parameters such as irradiation time, initial dye concentration, and solution pH. In contrast, achieving high degradation efficiencies under lower-energy irradiation (UVB, UVA, or visible light) generally requires the participation of highly ROS, commonly generated in advanced oxidation processes (AOPs) such as UV/H_2_O_2_, heterogeneous photocatalysis, or the photo-Fenton process. These observations are further supported by the study of Abbas, who reported the efficient direct photolysis of the structurally related sulfone phthalein dye bromophenol blue under UVC irradiation, with degradation rates increasing markedly under alkaline conditions as a consequence of changes in the dye speciation [18].

### 2.2. Enzymatic Treatment

It is well established that enzymatic reactions are influenced by several parameters, including temperature, pH, and enzyme and substrate concentrations [19].

To evaluate the enzymatic degradation of BTB by laccase of *Trametes versicolor* (L*Tv*), total soluble protein contents in the L*Tv* preparation (determined by Bradford) ranging from 1.4 to 3.5 μg mL^−1^ were tested, while maintaining a fixed BTB concentration (~25 μg mL^−1^).

Considering that typical fungal laccases are generally stable and catalytically active toward a broad range of substrates within a temperature interval of 20–50 °C and exhibit optimal activity in the pH range of 4–7 [20,21], the assays were conducted at pH 5, 6, and 7 and at temperatures of 20, 30, and 40 °C.

In each experimental series, all parameters were kept constant except for the variable under investigation. Under each condition, the apparent enzymatic degradation constant (*k*_ENZ_) was determined using Equation (1) by monitoring changes in the dye absorption spectrum as a function of reaction time.

**Figure 4:**
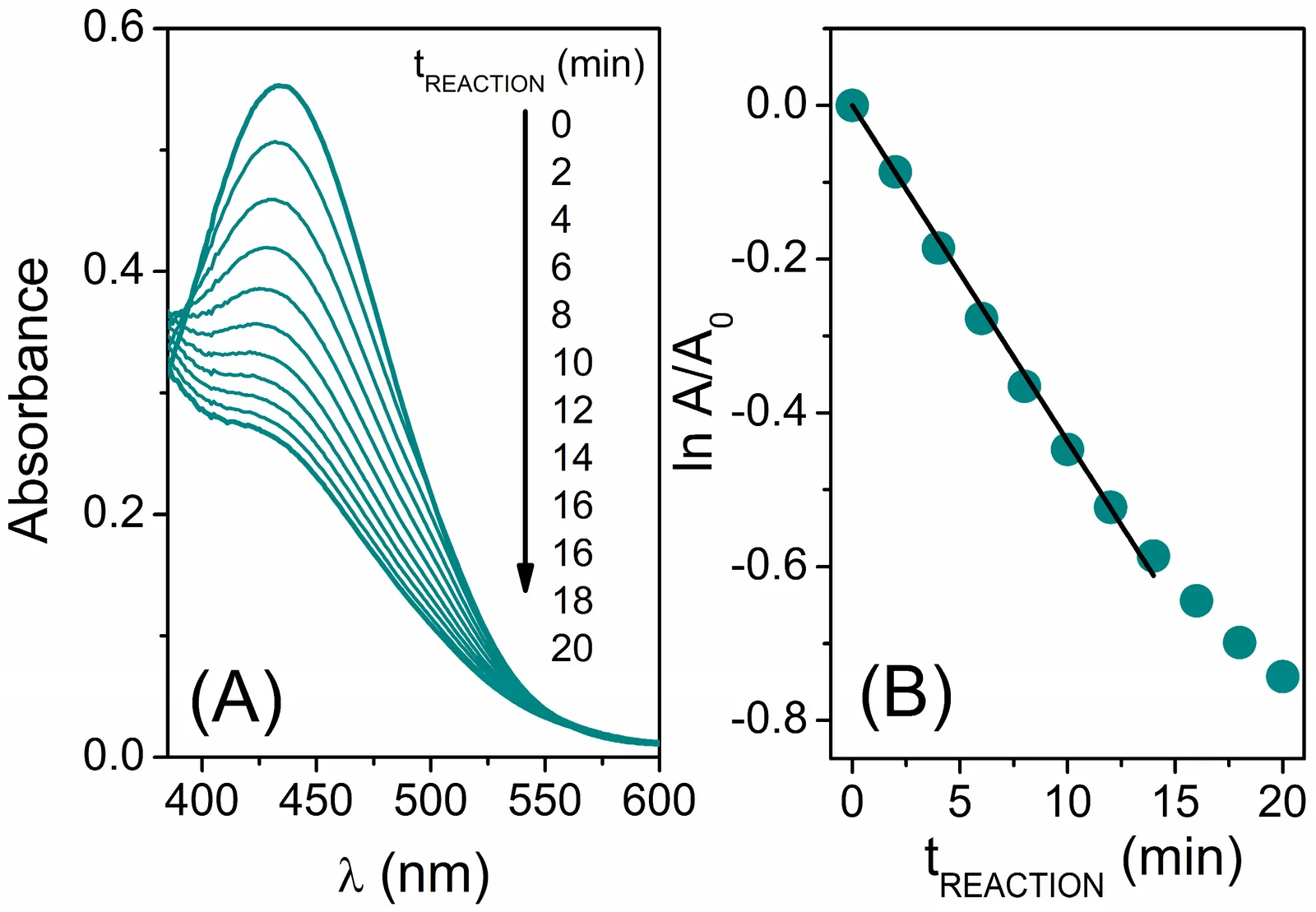
(**A**) Evolution of the absorption spectrum of BTB (25 μg mL^−1^) in the presence of 3.5 μg mL^−1^ of L*Tv* at pH 5 and 40 °C. (**B**) Graphic representation of Equation (1) used for the determination of the apparent kinetic enzymatic degradation constant, *k*_ENZ_; the solid line represents the linear regression of the experimental data.

Representative results are shown in Figure 4. Figure 4A illustrates the evolution of the absorption spectrum of BTB in the presence of 3.5 μg mL^−1^ of L*Tv* at pH 5 and 40 °C. The pronounced decrease in dye absorbance clearly demonstrates enzymatic degradation under the selected conditions.The corresponding logarithmic plot used to determine *k*_ENZ_ from the slope is shown in Figure 4B.

**Figure 5:**
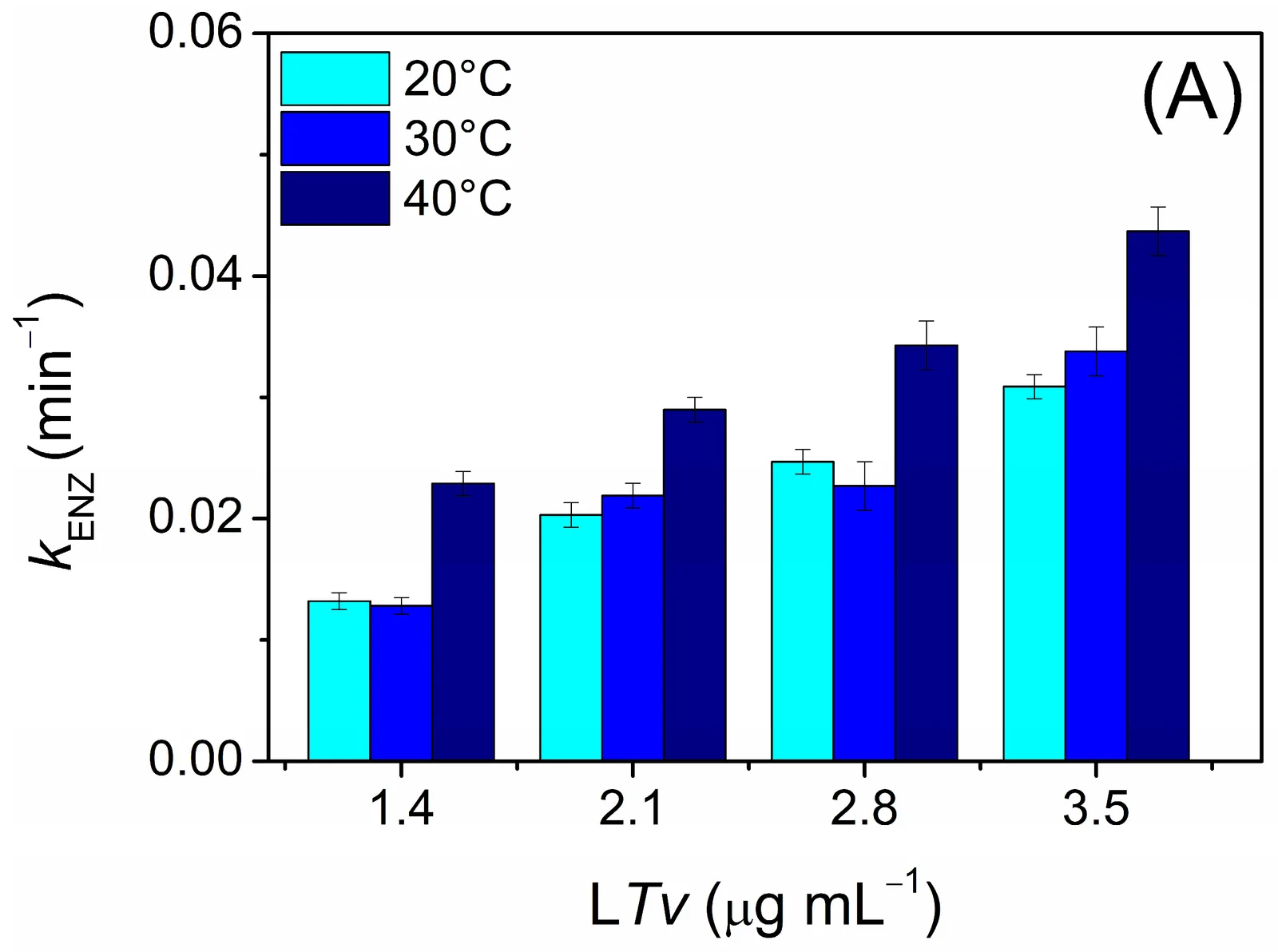

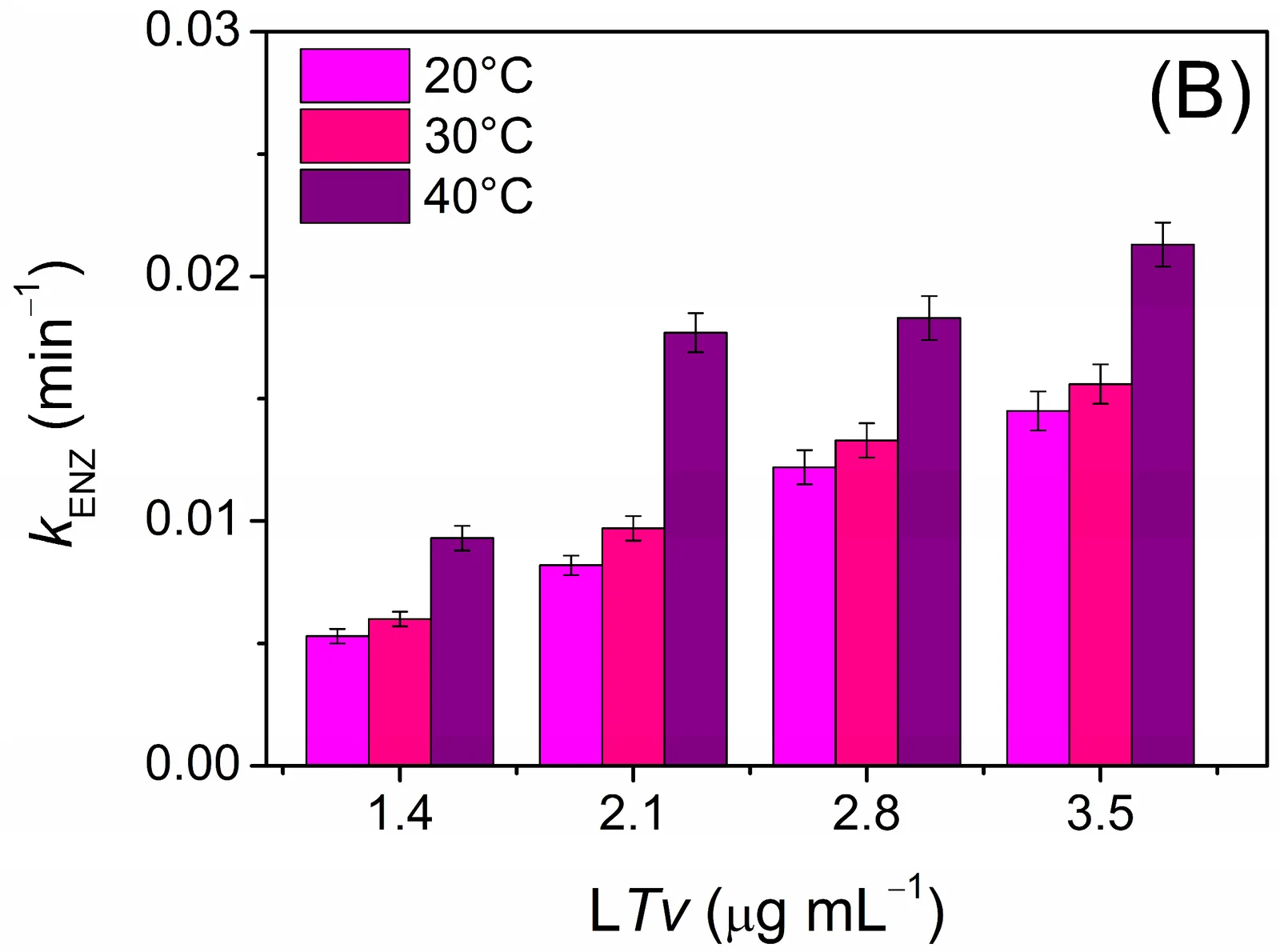
Apparent enzymatic degradation constants (*k*_ENZ_) of BTB as a function of total soluble protein content in the L*Tv* preparation (determined by the Bradford assay) and reaction temperatures at (**A**) pH 5 and (**B**) pH 6. Error bars represent ±1 standard deviation (SD) calculated from triplicate measurements.

To facilitate comparison among the evaluated variables, the *k*_ENZ_ values are summarized in the bar graphs shown in Figure 5A,B for pH 5 and 6, respectively.

The pH 7 condition is not included in Figure 5 because no measurable changes in the BTB absorption spectra were observed at any of the enzyme concentrations or temperatures tested, thus preventing calculation of kinetic constants. This result suggests that, under the studied conditions, BTB is not enzymatically degraded by L*Tv* at pH 7. A representative experiment performed at pH 7 using 3.5 μg mL^−1^ of L*Tv* and 40 °C is provided in the Supplementary Materials (Figure S2).

Analysis of the *k*_ENZ_ values reveals the following trends. At fixed temperature and pH, the enzymatic degradation rate of BTB increases with increasing L*Tv* content.

Likewise, at a constant amount of enzyme, *k*_ENZ_ increases with temperature, particularly at 40 °C, and also increases as pH decreases. According to the kinetic data, the optimal degradation conditions within the evaluated range correspond to pH 5 and 40 °C at the highest enzyme concentration tested.

It is widely recognized that increasing temperature generally enhances enzymatic reaction rates [19,22], as higher thermal energy promotes molecular motion and increases the frequency of effective enzyme–substrate collisions. This effect likely accounts for the observed increase in *k*_ENZ_ with temperature. However, excessively high temperatures may reduce enzymatic activity due to protein denaturation [19,22].

Regarding pH, enzyme activity is expected to decline when the reaction medium deviates from the optimal pH range. Changes in pH can alter the ionization state of acidic and basic amino acid residues, thereby modifying the enzyme’s conformation and active site geometry [19,22]. Such structural alterations may affect enzyme–substrate interactions and, consequently, the catalytic rate. Extreme pH conditions may additionally promote enzyme denaturation. In addition, the dependence of laccase activity on pH has been reported to become particularly relevant under neutral to alkaline conditions. Lloret et al. reported high removal efficiencies for several anti-inflammatory drugs and estrogenic hormones using laccase from *Myceliophthora thermophila* over the pH range of 4–7, whereas the removal efficiency decreased markedly at pH values above 7 [23]. Consistently, Kelbert et al. reported a marked decrease in the degradation of the anticancer drug doxorubicin by *Trametes versicolor* laccase at pH 8 and above [24]. This pH-dependent decrease in activity was attributed to the formation of hydroxide ions (OH^−^), which can bind to the T2/T3 copper cluster of the catalytic site. The coordination of hydroxide to these copper centers interferes with the internal electron transfer from the T1 copper to the trinuclear T2/T3 cluster, thereby disrupting the catalytic cycle and reducing laccase activity.

These observations could explain the variation in the *k*_ENZ_ values obtained for BTB at different pH values (5–7). Likewise, the decrease in enzymatic dye degradation, particularly the absence of detectable degradation at pH 7, could also be related to changes in the predominant chemical species of BTB as a function of pH.

**Figure 6:**
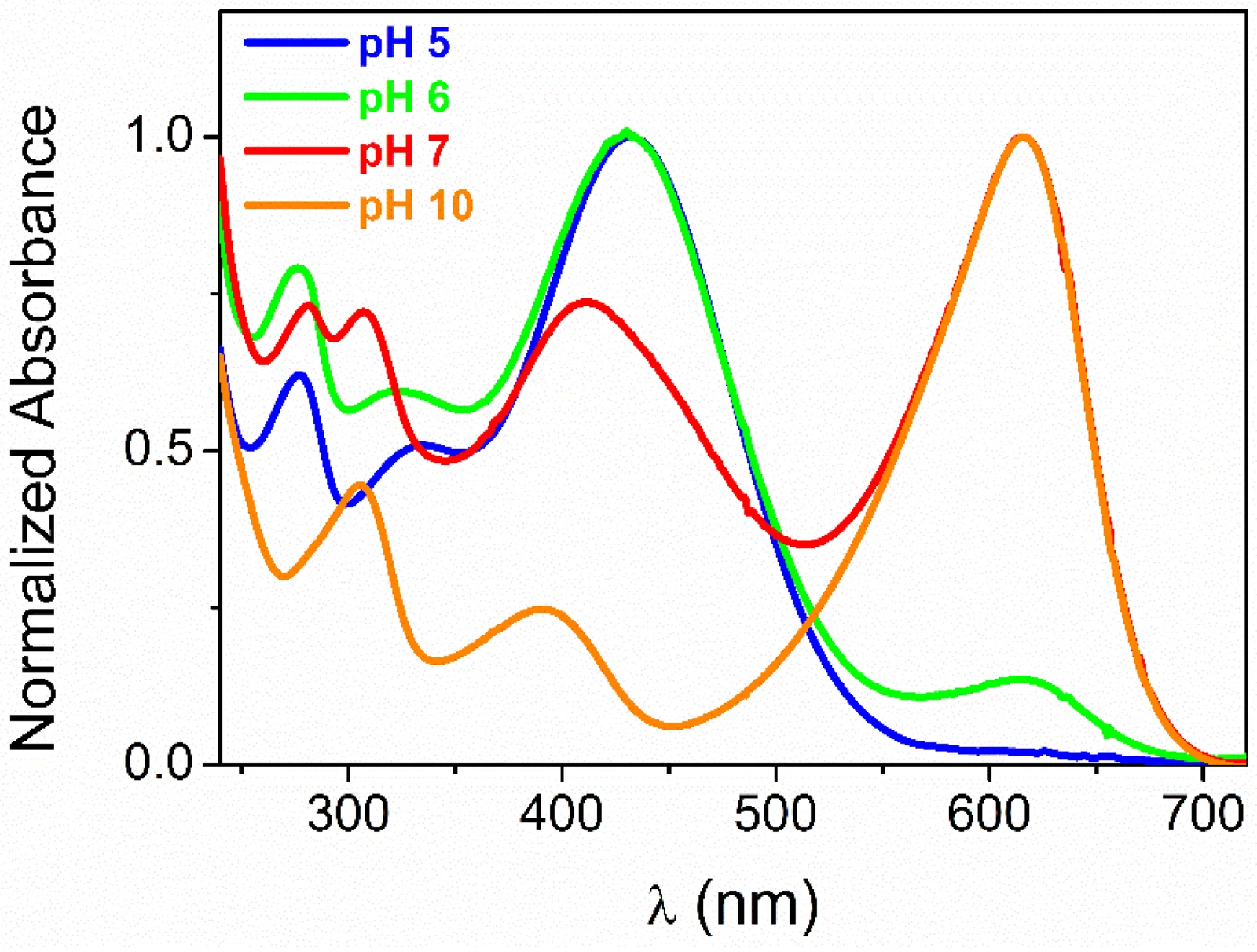
Normalized absorption spectra of BTB in buffer solutions at pH 5, 6, 7, and 10.

As previously noted, at pH values below its pK_a_ (~7.5), BTB predominantly exists as a monovalent anion; therefore, this species is expected to dominate at pH 5 and 6. As the pH approaches the pK_a_, however, the divalent anionic form becomes increasingly significant and predominates under alkaline conditions such as pH 10. Consequently, at pH 7, both the monovalent and divalent anionic forms of BTB are expected to be present in solution in appreciable proportions. This pH-dependent change in BTB speciation is also reflected in the normalized absorption spectra (at the wavelength of the maximum) shown in Figure 6.

The coexistence of two BTB species at pH 7 raised the possibility that L*Tv* might exhibit different affinities toward each form, potentially contributing to the reduced degradation observed under this condition.

To further investigate whether the absence of BTB degradation at pH 7 was related to the pH-dependent speciation of the substrate or to a loss of L*Tv* activity, a complementary assay was performed using 2,2′-Azino-bis(3-ethylbenzothiazoline-6-sulfonic acid) diammonium salt (ABTS). The use of ABTS allowed the effect of pH on L*Tv* catalytic activity to be evaluated independently of the relative abundance of the different BTB species present in the medium [25,26].

The reaction rates of L*Tv*-catalyzed ABTS oxidation were determined at different pH values, using the formation of the ABTS radical cation (ABTS^•+^) as a measure of enzymatic activity (absorbance at 420 nm) [26]. The results are presented in Figure S3 of the Supplementary Materials. A significant decrease in the rate of product formation was observed as the pH increased from 5 to 6, with no measurable activity detected at pH 7. These results suggest that the absence of BTB degradation at pH 7 is primarily associated with the marked loss of L*Tv* catalytic activity under this condition, rather than being solely attributable to changes in the relative abundance of BTB ionic species. Thus, although the pH-dependent speciation of BTB may influence enzyme–substrate interactions and probably contribute to the observed differences in BTB degradation kinetics, the results obtained with ABTS demonstrate that the reduced enzymatic activity of L*Tv* at pH 7 provides a sufficient explanation for the lack of BTB degradation. Additionally, the ABTS oxidation assay was performed at pH 10, where, as at pH 7, no detectable formation of ABTS^•+^ was observed, indicating the absence of measurable L*Tv* activity under this condition (Figure S3, Supplementary Materials).

Finally, with respect to enzyme content, it is well established that increasing enzyme concentration enhances reaction rate provided that sufficient substrate is available for binding; once the substrate becomes limiting, further increases in enzyme concentration do not result in higher reaction rates [19]. In the present study, an increase in dye degradation rate was observed as total soluble protein contents in the L*Tv* preparation increased. These results suggest that, under the experimental conditions employed, the optimal enzyme content for BTB degradation is likely equal to or greater than 3.5 μg mL^−1^. Within the evaluated concentration range, no evidence of saturation kinetics associated with complete enzyme–substrate interaction was detected.

It is well established that microbial laccases, particularly those of fungal origin, exhibit a high capacity for the decolorization and degradation of synthetic dyes commonly found in industrial effluents [27,28]. Specifically, white-rot fungi such as *Trametes versicolor* produce laccases with high redox potential, enabling the oxidation of recalcitrant aromatic compounds [27,28]. Accordingly, numerous studies have demonstrated the efficient transformation of dyes belonging to different chemical families such as Reactive Blue 19, Acid Orange 7, Malachite Green, Methylene Blue, Crystal Violet, Brilliant Green, and Indigo Carmine, through laccase-catalyzed processes [27,28,29,30]. In many cases, high decolorization efficiencies have been reported under mildly acidic conditions (pH 3–5) and temperatures between 30 and 50 °C [28,30], which is consistent with the conditions under which high reaction rates were observed in the present study for the degradation of BTB.

The degradation process is driven by redox reactions in which laccases oxidize dye molecules via electron transfer from the substrate to the enzyme (T1 copper site), and ultimately to molecular oxygen, generating lower-molecular-weight products with reduced chromaticity [28]. Typically, after the enzymatic reaction, free radicals are formed which lead to non-enzymatic radical reactions, such as polymerization and degradation [20,30]. The efficiency of this process is influenced not only by operational parameters, including pH, temperature, initial concentrations of the dye, availability of active enzyme and presence of oxygen, but also by the structural characteristics of the dye molecules. In particular, for aromatic substrates susceptible to laccase oxidation (e.g., aromatic amines, phenols and polyphenols), substituents located at ortho or para positions generally facilitate enzymatic oxidation compared with meta-substituted analogues by stabilizing the phenoxy or amino radical intermediates. On the other hand, strong electron-withdrawing groups tend to decrease electron density on the aromatic ring and increase resistance to oxidation, whereas electron-donating groups enhance susceptibility to enzymatic degradation [20,27,30].

### 2.3. Combined Treatment

In some cases, certain contaminants are difficult to efficiently degrade through a single treatment process, either due to their chemical stability or to the formation of persistent intermediates. For this reason, the study of combined processes has gained increasing attention and could represent a promising strategy for improving the removal of recalcitrant pollutants in the environment. Partially transformed compounds generated during one treatment step may exhibit higher reactivity toward other degradation mechanisms, allowing subsequent processes to proceed more efficiently. Combination between different treatment technologies can enhance the overall degradation efficiency and may also influence the toxicity of the final reaction products.

In this context, based on the kinetic information obtained from the individual photochemical and enzymatic treatments in our research, the combined process was investigated using the conditions that proved optimal for BTB degradation.

As previously discussed, the photochemical degradation of BTB was efficient under UVC irradiation, regardless of the presence of oxygen in the medium, and particularly under alkaline pH conditions. In contrast, in the presence of L*Tv*, optimal degradation was achieved at pH 5 and 40 °C, using catalyst content equal to or higher than 3.5 μg mL^−1^.

Considering these results, and taking into account that (a) the enzyme did not promote BTB degradation at pH 7 and may undergo inactivation at pH 10 (see explanation in Section 2.2 and Figure S3), and (b) the dye species present at pH 6 is similar to that prevailing at pH 5, suggesting that the photochemical response would be comparable (see Table S1), all experiments described in this section were conducted at pH 5.

Moreover, the enzymatic and photochemical treatments were performed sequentially rather than simultaneously, as direct exposure of laccase in aqueous solution to UV irradiation can result in a rapid loss of enzymatic activity. Our previous work demonstrated the sensitivity of L*Tv* in aqueous solution to UVB irradiation and the associated loss of enzymatic activity [31]. Similar UV-induced inactivation has also been reported for other oxidative enzymes, including polyphenol oxidase and peroxidase, under UVC irradiation [32]. Further support for the sequential approach was provided by Shi et al. [33], who directly compared simultaneous and sequential photolysis-laccase treatments for the degradation of dichlorophen and found the simultaneous process to be less efficient than the sequential treatment, which was attributed to partial enzyme inactivation under UV irradiation.

Accordingly, no chemical agent was added to quench the enzymatic activity prior to irradiation in the sequential enzymatic–photochemical treatment, as the subsequent UVC exposure was expected to rapidly inactivate any residual laccase activity.

The sequential treatments are summarized as follows:

Sequence 1:

BTB + UVC → (product)_PHOTOCHEMICAL_                        step 1

(product)_PHOTOCHEMICAL_ + L*Tv* → (product)_PHOTOCHEMICAL→ENZYMATIC_       step 2

Sequence 2:

BTB + L*Tv* → (product)_ENZYMATIC_                            step 1

(product)_ENZYMATIC_ + UVC → (product)_ENZYMATIC→PHOTOCHEMICAL_          step 2

**Figure 7:**
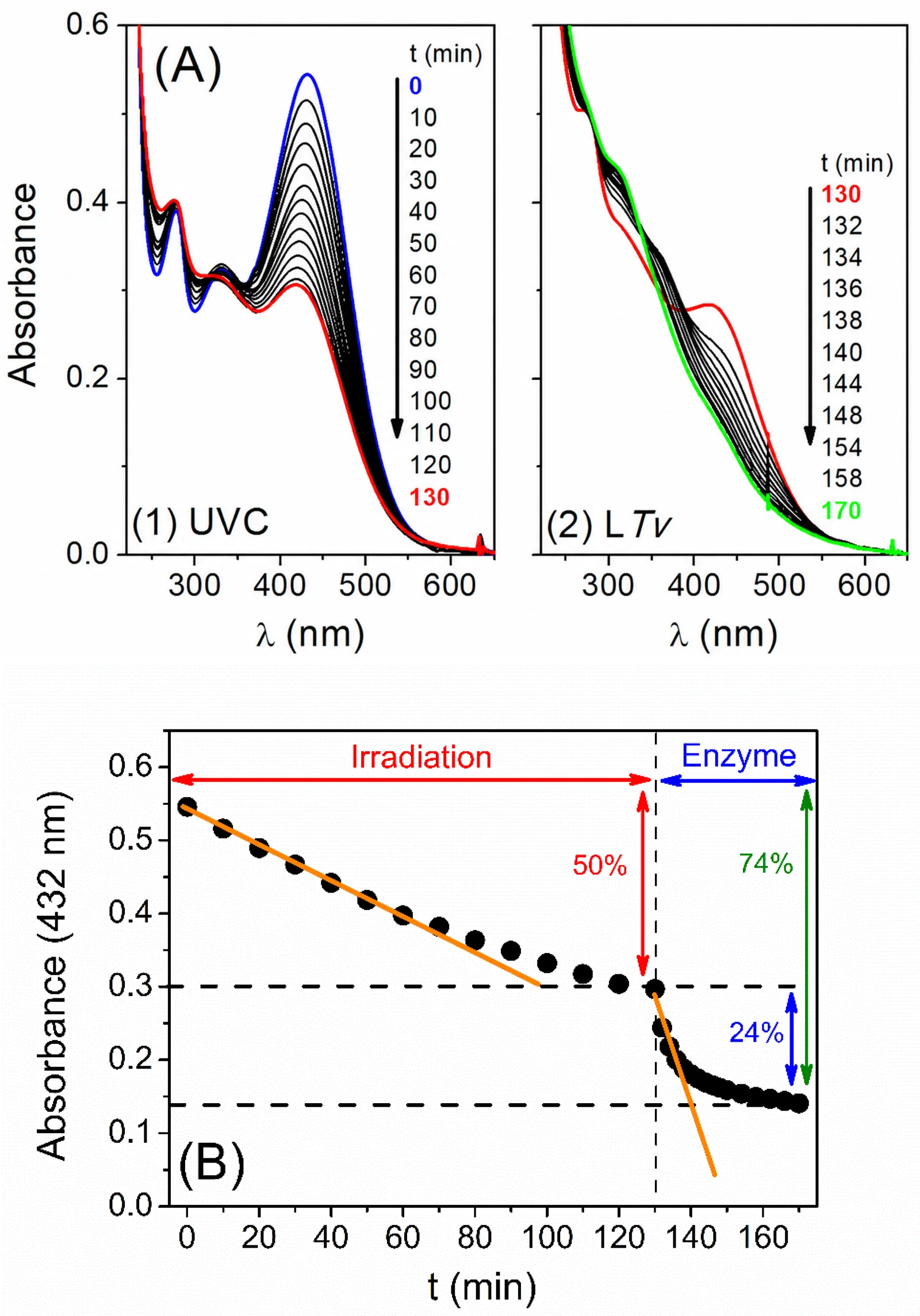
(**A**) Evolution of the absorption spectrum of BTB during photochemical treatment (UVC irradiation) at pH 5 under air atmosphere (1) and subsequent enzymatic treatment with 3.5 μg mL^−1^ of L*Tv* at pH 5 and 40 °C (2). (**B**) Variation of BTB absorbance at 432 nm during the combined treatment following the photochemical–enzymatic sequence. Linear regressions (solid lines) were fitted to each stage of the process to emphasize the change in slope between the initial rapid degradation and the subsequent slower regime. The green arrow indicates the overall percentage of BTB degradation achieved after completion of the combined treatment.

**Figure 8:**
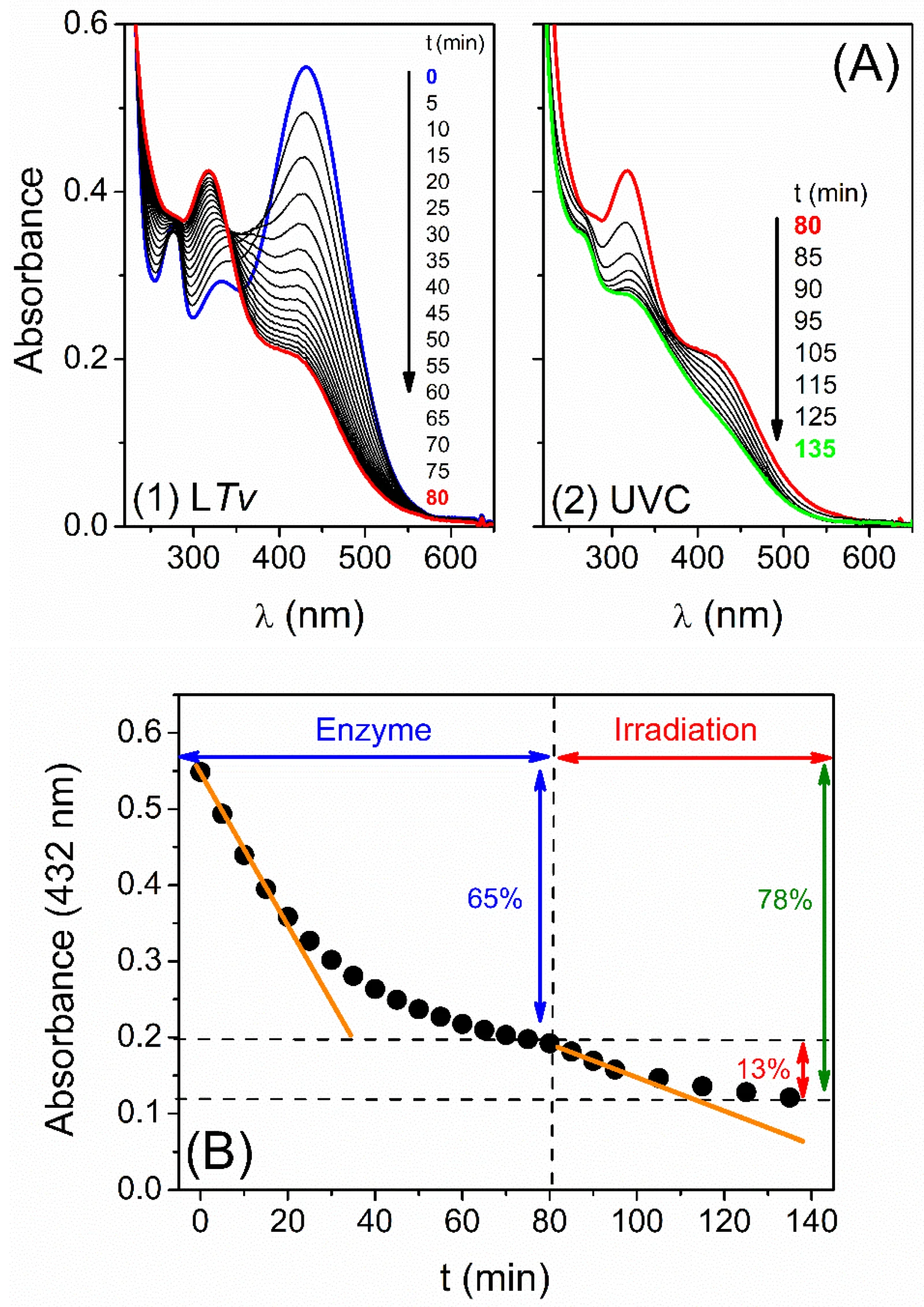
(**A**) Evolution of the absorption spectrum of BTB during enzymatic treatment with 3.5 μg mL^−1^ of L*Tv* at pH 5 and 40 °C (1), followed by photochemical treatment (UVC irradiation) at pH 5 under air atmosphere (2). (**B**) Variation of BTB absorbance at 432 nm during the combined treatment following the enzymatic–photochemical sequence. Linear regressions (solid lines) were fitted to each stage of the process to emphasize the change in slope between the initial rapid degradation and the subsequent slower regime. The green arrow indicates the overall percentage of BTB degradation achieved after completion of the combined treatment.

For both treatment sequences, the duration of the first step was determined from the absorbance decay profile at 432 nm, the wavelength used to monitor BTB degradation. Initially, a well-defined negative slope was observed, corresponding to the efficient degradation of the parent dye. As the reaction progressed, however, the slope gradually decreased, approaching a plateau. This behavior could be attributed to the accumulation of transformation products that retain partial absorbance at 432 nm and are degraded less efficiently by the individual treatment (photochemical or enzymatic). Consequently, the transition to the second treatment step was established at the onset of this marked decrease in the degradation rate, allowing the complementary process to further transform the remaining compounds. The results obtained for each sequential treatment are presented in Figure 7 and Figure 8, where BTB degradation is expressed as percentage removal calculated according to Equation (2).

When the dye solution was initially treated by UVC irradiation for 130 min, a degradation of 50% was achieved, corresponding to the point at which no further significant changes in absorbance at 432 nm were observed. Subsequently, upon initiation of the enzymatic treatment, the degradation process resumed, leading to an additional 24% decrease in dye absorbance and a total degradation of 74% after 170 min.

A similar behavior was observed when the sequence was initiated with the enzymatic process. After 80 min of treatment with L*Tv*, BTB degradation reached 65%. Thereafter, exposure of the remaining solution to photochemical treatment resulted in an additional degradation of 13%, reaching a total dye removal of 78% after 140 min.

As can be observed, although the individual processes are highly efficient and lead to substantial degradation within relatively short reaction times, they are not sufficient to achieve complete dye removal. However, the incorporation of a second treatment step enhances the overall performance of the system. While complete degradation of BTB was not achieved (as evidenced by the residual absorbance signal), the sequential treatments resulted in overall degradation values exceeding 70% for both experimental sequences.

The improved performance observed for the sequential treatments suggests a complementary action between the photochemical and enzymatic processes. UVC irradiation likely induces partial structural modifications of the dye molecule, generating products that may be more amenable to enzymatic oxidation by laccase. Conversely, the enzymatic step may also contribute to the transformation of photogenerated compounds, allowing the degradation process to proceed further.

The spectral evolution observed during the two sequential treatments provides additional qualitative evidence of differences in the species formed and subsequently transformed during each sequence (Figure 7A or Figure 8A). In the photochemical–enzymatic sequence (Figure 7A), the initial UVC treatment progressively decreases the main BTB absorption band and the absorption at shorter wavelengths, leaving only a weak and broad residual signal in the 300–400 nm region at the end of the photochemical stage (Figure 7A(1)). Upon subsequent treatment with L*Tv* (Figure 7A(2)), this residual absorption continues to decrease, without a clear accumulation of a new broad absorption band. This behavior suggests that the residual photoproducts formed during UVC irradiation are further transformed during the enzymatic step, although their chemical nature cannot be established from the UV–Vis spectra.

In contrast, the enzymatic–photochemical sequence (Figure 8A) exhibits a more evident spectral change during the first treatment stage. During laccase treatment (Figure 8A(1)), the progressive decrease of the main BTB band is accompanied by the development of a broad and less structured absorption in the 300–400 nm region. Although this feature cannot be assigned to specific compounds based on UV–Visible data alone, it is consistent with the accumulation of transformation products with lower chromophoric character than the parent dye, potentially including oxidized or otherwise structurally modified aromatic species generated during laccase-mediated oxidation [20,27,30]. When the solution is subsequently subjected to UVC irradiation (Figure 8A(2)), this residual absorption progressively decreases, suggesting further transformation and removal of these species. Thus, the two sequences display different spectral patterns: whereas the photochemical–enzymatic sequence shows a progressive attenuation of the residual absorption after UVC treatment, the enzymatic–photochemical sequence shows a more evident accumulation of intermediate species during the enzymatic stage followed by their subsequent transformation under UVC irradiation.

Taken together, these spectral changes are consistent with the sequential formation and removal of transformation products and provide qualitative evidence for the complementary roles of the two treatments. However, further characterization by techniques such as LC-MS/MS or NMR would therefore be required to confirm the chemical nature of the formed intermediates, the corresponding transformation pathways, and the relationships between specific transformation products and the effects observed in the ecotoxicity tests discussed in Section 2.4.

In a previous study, Karimi et al. [34] investigated the efficiency of AOPs, enzymatic treatments, and their sequential combinations for the remediation of colored effluents from chemithermomechanical pulp (CMP) and paper mills. In that investigation, the fungal enzymes laccase from *Trametes versicolor* and versatile peroxidase from *Bjerkandera adusta* were applied, and the influence of an external mediator (ABTS) on enzymatic degradation was also assessed. Additionally, the authors evaluated the effect of the Fenton and Photo-Fenton processes on the decolorization of the effluents. The results found in this work indicated that both enzymes were able to effectively decolorize the dark brown effluent to a light-yellow transparent solution, with improved decolorization rates in the presence of ABTS. Additionally, the photo-Fenton process showed a considerable effect on color removal. The analysis of sequential treatments (biological-chemical and chemical-biological) revealed that combined approaches achieved higher decolorization efficiencies than individual treatments, with the enzymatic step followed by a photo-Fenton post-treatment providing the best performance for CMP effluents.

Moreover, Shi et al. [33] demonstrated that the combination of photolytic processes under simulated solar irradiation and laccase catalysis can mutually enhance the degradation of dichlorophen in aqueous solution. Notably, the sequential application of the two processes resulted in better treatment performance than either individual process, leading to lower toxicity of the resulting products.

In accordance with these findings, the results obtained in our study also suggest that combining treatments can improve the transformation of the target contaminant and likely influence the nature of the degradation products, highlighting the potential advantages of integrating pollutant removal processes.

To place the present results in the context of previous studies on BTB degradation in aqueous media, a comparison with selected literature reports is provided in Table S2 (Supplementary Materials).

The table summarizes the main treatment approaches, experimental conditions, and reported BTB removal or degradation efficiencies. Because substantial differences exist among studies in terms of initial BTB concentration, pH, irradiation conditions, oxidant or catalyst dosage, and reaction configuration, the reported efficiencies should be interpreted as comparative rather than directly equivalent performance indicators.

### 2.4. Ecotoxicological Analysis

Although the combined treatments led to higher degradation percentages than the individual processes, the decrease in dye absorbance does not necessarily imply a reduction in the toxicity of the resulting transformation products. Indeed, partial degradation processes may generate compounds whose environmental impact differs from that of the parent molecule. Furthermore, based on the spectral modifications observed for the two sequences (see Figure 7A or Figure 8A), the degradation products formed may differ in nature and could represent distinct environmental risks. Therefore, in order to evaluate the environmental relevance of the treatments investigated in this work, the toxicity of the products formed during the individual and combined processes was subsequently assessed.

The bioluminescence inhibition bioassay has been widely applied for toxicity monitoring and for obtaining rapid information on the toxic or ecotoxic potential of environmental samples. Its extensive use is mainly attributed to several advantages, including short test duration, high sensitivity, cost-effectiveness, and ease of operation. Moreover, this bioassay has proven to be applicable to a wide variety of matrices, including wastewater, river water, sewage sludge, landfill leachate and treated effluents, among others, making it a versatile tool for environmental toxicity assessment [35,36].

The assay is based on the marine Gram-negative bacterium *Vibrio fischeri* (formerly referred to as *Photobacterium phosphoreum*), whose bioluminescence is directly linked to the metabolic activity of the bacterial cells. Consequently, the presence of toxic substances that interfere with cellular metabolism leads to the inhibition of enzymatic activity and, therefore, to a measurable decrease in light emission [35].

In the present work, the inhibition of bioluminescence was evaluated and expressed as the percentage of luminescence inhibition (% INH) for BTB samples treated by individual and combined photochemical and enzymatic processes, considering the two treatment sequences described in the previous section.

**Figure 9:**
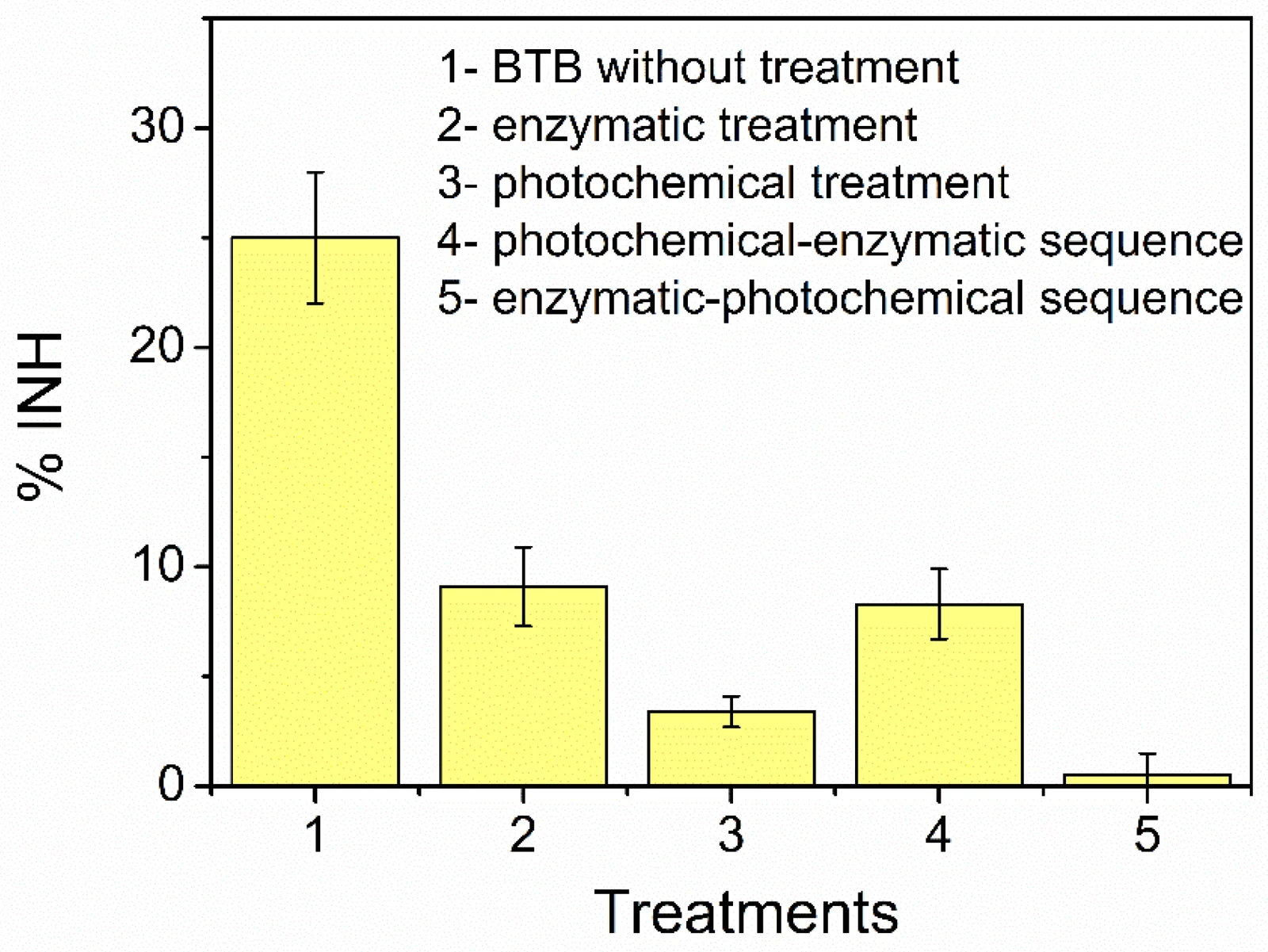
Percentage inhibition of *Vibrio fischeri* bioluminescence (% INH) in the presence of untreated BTB and after individual and sequential combined treatments. Toxicity assays were performed after 5 min incubation with the samples (1/4 diluted). Error bars represent ± 1 standard deviation (SD) calculated from triplicate measurements.

Figure 9 clearly shows that the untreated dye exhibited a significantly higher level of toxicity than the products generated by any of the evaluated treatments. These results demonstrate that both UVC irradiation and enzymatic catalysis, applied individually or in combination, significantly reduced the toxicity of the parent compound.

Interestingly, treatments including a photochemical stage consistently yielded products with lower toxicity than the enzymatic treatment alone. Moreover, the order in which both processes were combined affected the final toxicity. Applying UVC irradiation after the enzymatic treatment produced the lowest % INH, whereas the reverse sequence resulted in some residual toxicity.

These observations suggest that the photochemical step plays an important role in transforming potentially toxic byproducts generated during enzymatic degradation into less harmful compounds. This interpretation is consistent with the findings of Shi et al. [33], who identified photolysis as a crucial step for the removal of toxic oxidation products formed during laccase-catalyzed degradation of dichlorophen.

Importantly, the enzymatic–photochemical sequence (Sequence 2) not only led to the lowest residual toxicity, with approximately 1% INH, but also showed the highest overall degradation efficiency and the shortest total treatment time, compared with Sequence 1 (see Section 2.3). Thus, the sequence that provided the greatest removal of BTB also generated products with the lowest residual toxicity, making it the most attractive candidate for practical implementation.

A comprehensive comparison of the individual and combined treatments, including treatment conditions, reaction times, degradation efficiencies and ecotoxicological indices, is provided in Table 1, facilitating an integrated assessment of their overall performance.

Our findings highlight the importance of complementing degradation studies with toxicity assessment, demonstrating that efficient pollutant removal should be accompanied by the formation of less toxic transformation products than the parent compound.

## 3. Materials and Methods

### 3.1. Chemicals

Bromothymol blue (BTB, 95%) was purchased from Sigma-Aldrich (Buenos Aires, Argentina). Laccase (E.C. 1.10.3.2) from *Trametes versicolor* (L*Tv*, 0.5 U mg^−1^; 1 U defined as the amount of enzyme that converts 1 μmol of catechol per minute at pH 5.0 and 25 °C) was also obtained from Sigma-Aldrich and used without further purification.

2,2′-Azino-bis(3-ethylbenzothiazoline-6-sulfonic acid) diammonium salt (ABTS, ≥98%) was provided from Sigma-Aldrich. The specific reactive oxygen species (ROS) scavenger’s superoxide dismutase (SOD) from bovine erythrocytes and catalase from bovine liver (CAT) were purchased from Sigma-Aldrich, while Sodium azide 99% (NaN_3_) and isopropyl alcohol 99.7% (IPA) were purchased from Merck (Buenos Aires, Argentina). These reagents were used as received.

All experiments were performed in buffer solutions prepared with triple-distilled water according to a previously reported procedure [37]. The buffer pH was adjusted using Na_2_HPO_4_, KH_2_PO_4_, NaHCO_3_ and NaOH (Cicarelli, analytical grade, San Lorenzo, Santa Fe, Argentina).

### 3.2. Experimental Procedures

#### 3.2.1. Kinetic Analysis of Individual Processes

Steady-state photolysis experiments were conducted by direct irradiation of BTB solutions in buffered media using a Rayonet photochemical reactor (Rayonet Ltd., Branford, CT, USA) equipped with UV lamps. Irradiation was performed in the UVC, UVB, and UVA regions using lamps with emission maxima at 254 nm, 300 nm and 364 nm respectively. In each case, three lamps were employed. The irradiances of the lamps, measured on the surface of the quartz cell located in the center of the photoreactor, were 4800 μW/cm^2^, 4700 μW/cm^2^ and 2600 μW/cm^2^ for the lamps with maximum emission at 254 nm, 300 nm and 364 nm, respectively. The irradiance measurements were performed using a short-wave UVC light meter (VTSYIQI handheld, Hefei, Anhui, China) and a UV radiometer (UV-340A, Lutron Electronic Enterprise Co., Ltd., Taipei, Taiwan). The photoreactor is equipped with a ventilation system to minimize temperature fluctuations during irradiation.

Visible-light photodegradation experiments were performed using a home-made photoreactor equipped with a 150 W quartz-halogen lamp as the light source, a thermal filter to remove infrared radiation, and appropriate cut-off filters to eliminate UV contributions.

Before each irradiation experiment, the light source was switched on and allowed to reach stable emission for 15 min prior to sample irradiation. A schematic of the irradiation systems employed is shown in Figure S4. All photochemical experiments were carried out at room temperature (25 ± 2 °C).

Photochemical and enzymatic degradation processes, evaluated either individually or sequentially, were monitored by recording UV–Visible absorption spectra using a Hewlett Packard 8453A diode-array spectrophotometer (Hewlett Packard, CA, USA) and a standard quartz cuvette with a 10 mm optical path length and 3 mL volume.

Changes in BTB (25 μg mL^−1^) absorbance upon irradiation or enzymatic treatment were analyzed assuming pseudo-first-order kinetics according to Equation (1):

(1) $$\ln\frac{A}{A_{0}}=\;-k_{app}\;t$$

where *A*_0_ and *A* represent the absorbance of BTB at the selected monitoring wavelength at time zero and at reaction time, *t*, respectively, and *k*_app_ is the apparent pseudo-first-order rate constant obtained from the initial slope of the plot ln(*A*/*A*_0_) versus *t* (min). The monitoring wavelength was set at 432 nm for pH ≤ 6 and at 615 nm for pH 10. Data points were collected for a sufficient period to monitor BTB consumption until the absorbance-versus-time profile deviated from linear behavior, corresponding to 15–30 min for the individual irradiation and enzymatic kinetic runs and 140–180 min for the combined treatments, based on the total duration of the two sequential steps.

To distinguish between degradation pathways, the apparent rate constant is denoted as *k*_PHOT_ for photochemical degradation and *k*_ENZ_ for enzymatic degradation.

To evaluate the influence of pH and dissolved oxygen on the photodegradation mechanism in the studied solutions, *k*_PHOT_ was determined at pH 6 and 10 under air- and argon-saturated conditions. For this purpose, dye solutions were irradiated either in equilibrium with air or in hermetically sealed cuvettes previously purged with argon for 20 min.

To investigate the involvement of different ROS in the photodegradation of BTB, *k*_PHOT_ values were determined in the absence and presence of specific ROS scavengers. For this purpose, dye solutions were irradiated in separate experiments with the addition 1 × 10^−7^ M of SOD, 1 × 10^−7^ M of CAT, 2 × 10^−2^ M of IPA, and 1 × 10^−2^ M of NaN_3_. The enzymes SOD and CAT were used for the detection of superoxide radical anion (O_2_^•−^) and hydrogen peroxide (H_2_O_2_), respectively [38,39]. Meanwhile, IPA and NaN_3_ were used for hydroxyl radical (HO^•^) and singlet oxygen (O_2_(^1^Δ_g_)) detection, respectively [38,40].

The effects of pH, temperature, and L*Tv* concentration for enzymatic degradation of BTB were investigated by determining *k*_ENZ_ at pH 5, 6, and 7; at 20, 30, and 40 °C; and at total soluble protein in the L*Tv* preparation ranging from 1.4 to 3.5 µg mL^−1^, while maintaining a constant dye concentration of 25 μg mL^−1^. In each set of experiments, only one parameter was varied while the others were kept constant. Temperature control during enzymatic kinetic measurements was achieved using a thermostatted cell holder.

For the complementary assay using ABTS (9 × 10^−5^ M) as substrate, 0.7 μg mL^−1^ of L*Tv* was used at pH 5, 6, 7, and 10 and 30 °C. The formation of the ABTS radical cation (ABTS^•+^) resulting from L*Tv*-catalyzed oxidation was monitored spectrophotometrically at 420 nm [26], and the initial reaction rate was determined from the linear increase in absorbance over time.

The total soluble protein content of the commercial L*Tv* preparation was determined by the Bradford assay using bovine serum albumin (BSA) as a standard. Absorbance was measured at 595 nm, and protein concentrations were estimated from a BSA standard curve [41].

#### 3.2.2. Cooperative Treatments

Combined experiments were performed under the optimal conditions identified for individual photochemical and enzymatic degradation processes and evaluated using two sequential approaches:

1. The BTB solution was first irradiated and subsequently treated with the enzyme.
2. The solution was initially incubated with L*Tv* and then subjected to irradiation.

In the first sequence, 25 μg mL^−1^ of dye solution was irradiated with UVC light under an air atmosphere. The resulting solution was then brought to 40 °C, after which laccase was added to reach a final protein content (determined by the Bradford assay) of 3.5 μg mL^−1^, and the enzymatic reaction was allowed to proceed at this temperature. In the second sequence, 25 μg mL^−1^ of dye solution was first equilibrated at 40 °C and treated with laccase (final content: 3.5 μg mL^−1^). The resulting solution was subsequently subjected to UVC irradiation under an air atmosphere.

The final pH was not independently measured at the end of each experiment; however, it was expected to remain close to the nominal value established by the buffer, given its buffering capacity and the submillimolar concentration of BTB employed in the experiments.

Each treatment step (photochemical or enzymatic) was continued until no significant changes in BTB absorbance were observed at the selected monitoring wavelength. Dye removal at each stage was quantified as percentage degradation using Equation (2):

(2) $$\%\;Degradation=\left(\frac{A_{0}-A_{t}}{A_{0}}\right)\times 100$$

where *A*_0_ and *A*_t_ correspond to the absorbance at maximum of absorption band, at time zero and at reaction time *t*, respectively.

#### 3.2.3. Ecotoxicological Assessment

The ecotoxicological impact of the treated BTB solutions was evaluated using the luminescent bacterial inhibition assay based on *Vibrio fischeri*. The test was performed following the recommendations described in the Microtox^®^ Acute Toxicity Basic Test Procedures Manual provided by Modern Water (New Castle, DE, USA).

All reagents were supplied as part of the Microtox^®^ Acute Toxicity kit (Modern Water) and included: the Acute Reagent, Reconstitution Solution, Diluent, and Osmotic Adjusting Solution (OAS). The Acute Reagent consists of a freeze-dried preparation of a selected strain of the marine bacterium *Vibrio fischeri* (formerly *Photobacterium phosphoreum*, NRRL B-11177).

The Diluent is a non-toxic 2% (*w*/*v*) sodium chloride (NaCl) solution used for diluting both the samples and the bacterial suspension (prepared in the Reconstitution Solution). This saline medium provides the osmotic protection required by the marine bacterium. The Osmotic Adjusting Solution (OAS) consists of a non-toxic 22% (*w*/*v*) NaCl solution used to adjust the osmotic pressure of the samples to approximately 2% NaCl prior to the assay. A bacterial suspension in 2% NaCl without the test sample was used as control.

All measurements were performed at 15 °C in duplicate. The luminescence emitted by *Vibrio fischeri* was measured using a conventional spectrofluorometer (FluoroMax-4 Horiba Instruments Incorporated, NJ, USA) operated with the excitation source switched off and recording the emitted light at 490 nm.

The toxic effect of the untreated and treated BTB solutions was expressed as the percentage of luminescence inhibition (%INH) according to Equation (3) [35,42].

(3) $$\%INH=\left(1-\frac{L_{t}}{L_{0}\;x\;f}\right)\times 100$$

where *L*_0_ is the initial luminescence intensity of the bacterial suspension measured immediately after the addition of the tested solution (diluted 1/4), and *L*_t_ is the luminescence intensity measured after the exposure time *t* (5 min incubation). The term *f* represents the time correction factor, which accounts for the natural decrease in bacterial luminescence during the assay and was calculated according to Equation (4):

(4) $$f=\frac{L_{c,0}}{L_{c,t}}$$

where *L*_c,0_ and *L*_c,t_ correspond to the luminescence intensity of the control (bacterial suspension without sample) measured at the initial time and after the exposure time, *t* (5 min incubation), respectively.

Taking into account the color of the sample’s solutions, %INH was corrected by their absorbances at 490 nm according to the method described elsewhere [43].

## 4. Conclusions

The present study demonstrated that bromothymol blue can be effectively degraded through both photochemical and enzymatic treatments, although their performance strongly depends on the operating conditions. Photochemical degradation was most effective under UVC irradiation and alkaline conditions, with the results being consistent with a predominantly unimolecular photodegradation pathway, whereas enzymatic degradation by *Trametes versicolor* laccase was favored at acidic pH, elevated temperature, and high total soluble protein content in the laccase preparation.

Sequential application of both treatments improved the overall degradation efficiency compared with the individual processes, yielding degradation values above 70% for both treatment sequences. These results indicate complementary processes between photochemical and enzymatic mechanisms, where transformation products generated during one step become susceptible to further degradation in the subsequent stage. From a practical perspective, however, the use of acidic conditions (pH 5) in the sequential treatments would require a neutralization step prior to discharge into receiving water bodies.

Importantly, ecotoxicological assays demonstrated that all treatments reduced the toxicity of the parent dye, with the lowest toxicity levels observed for treatments involving UVC irradiation. These findings highlight the importance of incorporating a photochemical step to minimize the environmental impact associated with transformation products.

Overall, the integration of photochemical and enzymatic treatments constitutes a promising strategy for the remediation of dye-contaminated waters, combining high degradation efficiency with reduced ecotoxicological risk. This approach provides a sustainable framework for the development of advanced treatment technologies for dye-containing wastewaters.

## Figures and Tables

**Table 1:** Summary of individual and combined treatments applied to 25 μg mL^−1^ BTB, consolidating data from Figure 3, Figure 5, Figure 7, Figure 8 and Figure 9.

Treatment	Experimental Condition	Reaction Time	Degradation (%)	% INH (Toxicity)
Untreated BTB	-	-	-	~25%
Photochemical (individual)	optimal for combinations: UVC, pH 5, air.	~30 min (kinetic run)	50%	~3%
Enzymatic (individual)	optimal for combinations: L*Tv* 3.5 µg mL^−1^, pH 5, 40 °C	~20 min (kinetic run)	65%	~9%
Combined-Sequence 1 (Photochemical→Enzymatic)	UVC (pH 5, air) then L*Tv* 3.5 µg mL^−1^ (pH 5, 40 °C)	130 min (step 1) + 40 min (step 2) = 170 min total	50% (step 1) + 24% (step 2) = 74% total	~8%
Combined-Sequence 2 (Enzymatic→Photochemical)	L*Tv* 3.5 µg mL^−1^ (pH 5, 40 °C) then UVC (pH 5, air)	80 min (step 1) + 60 min (step 2) = 140 min total	65% (step 1) + 13% (step 2) = 78% total	~1% (lowest)

## Data Availability

The original contributions presented in this study are included in the article and Supplementary Materials. Further inquiries can be directed to the corresponding authors.

## Supplementary Materials

The following supporting information can be downloaded at: https://www.mdpi.com/article/10.3390/ijms27177767/s1.
