# Supplementary material for: Individual and Cooperative Photochemical–Enzymatic Processes for the Degradation of the Dye Bromothymol Blue: Kinetic and Eco-Toxicity Analysis
Source: Int J Mol Sci. 2026 Aug 30;27(17):7767. doi: 10.3390/ijms27177767 (PMC13566542; doi:10.3390/ijms27177767)
Supplement: Supplementary file 1 [file ijms-27-07767-s001.zip › ijms-4481253-supplementary.pdf]

Supplementary Material

**Individual and Cooperative Photochemical-Enzymatic Processes for the Degradation of the Dye Bromothymol Blue. Kinetic and Eco-toxicity Analysis.**

Andrea S. Urquiza[1,2], Agustina Reynoso[1,2], M. Alicia Biasutti[1,3], Hernán A. Montejano *[1,2], Eugenia Reynoso *[1,3]

[1] Departamento de Química, Facultad de Ciencias Exactas, Físico-Químicas y Naturales, Universidad Nacional de Río Cuarto (UNRC), Ruta Nacional 36 Km 601, X5804BYA Río Cuarto, Córdoba, Argentina.

[2] Instituto de Investigaciones en Tecnologías Energéticas y Materiales Avanzados (IITEMA); CONICET – UNRC.

[3] Instituto para el Desarrollo Agroindustrial y de la Salud (IDAS); CONICET – UNRC.

*Corresponding authors. Tel. +54 358 467 6233.

e-mail address: hmontejano@unrc.edu.ar (H.A. Montejano), ereynoso@exa.unrc.edu.ar (E. Reynoso)

ORCID ID: Hernán A. Montejano: 0000-0002-8463-796X

Eugenia Reynoso: 0000-0002-7497-2887

**Table S1.** Apparent pseudo-first-order rate constants ($k_{PHOT}$; min$^{-1}$) for bromothymol blue (BTB) photodegradation under UVC and UVB irradiation at pH 6 and 10 in air- and argon-saturated solutions. The $k_{PHOT}$ value obtained under UVC irradiation at pH 5 in air is included for comparison.

| pH | Atmosphere | Irradiation | |
|---|---|---|---|
| | | UVC | UVB |
| 5 | Air | 0.0053 ± 0.0004 | - |
| 6 | Air | 0.0059 ± 0.0003 | 0.0012 ± 0.0002 |
| | Argón | 0.0099 ± 0.0005 | 0.0016 ± 0.0002 |
| 10 | Air | 0.063 ± 0.003 | 0.020 ± 0.001 |
| | Argón | 0.065 ± 0.003 | 0.022 ± 0.001 |

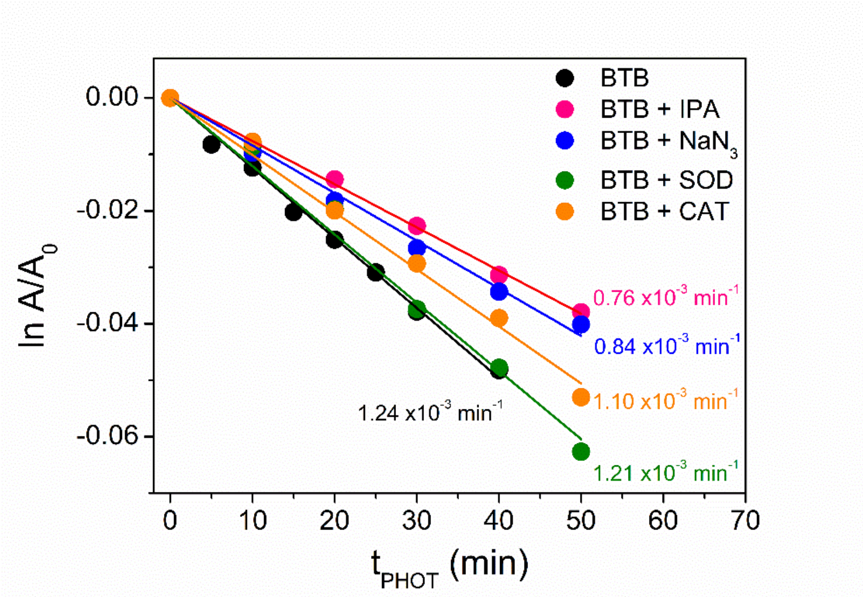


**Figure S1.** Pseudo-first-order kinetic plots used to determine the apparent rate constant for BTB photodegradation ($k_{PHOT}$, expressed next to each line of fit) from equation 1 in the absence (black circles) and in the presence of the following ROS scavengers: 2x10$^{-2}$ M isopropyl alcohol (IPA, pink circles), 1 x 10$^{-2}$ M sodium azide ($NaN_3$, blue circle), 1 x 10$^{-7}$ M superoxide dismutase (SOD, green circle), and 1 x 10$^{-7}$ M catalase (CAT, orange

circle).

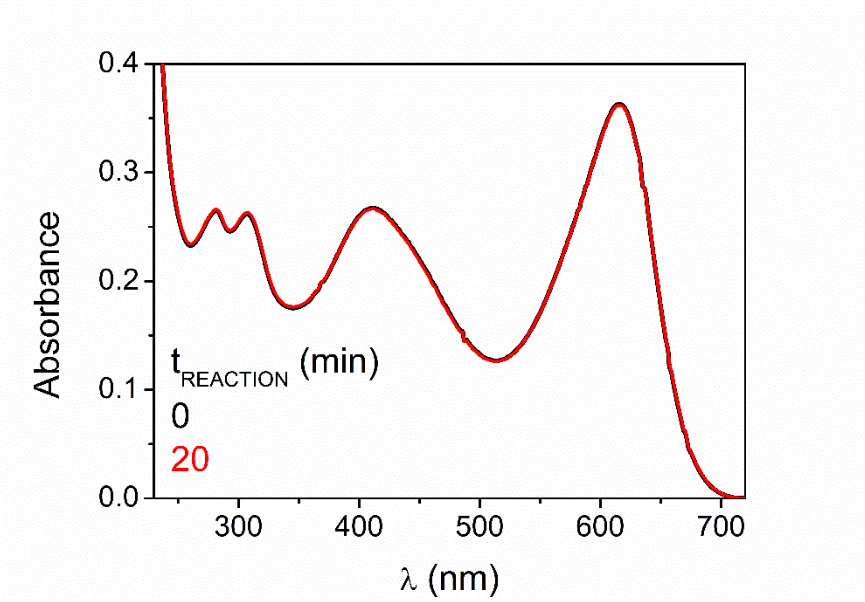


**Figure S2.** Evolution of the absorption spectra of BTB (25 μg mL$^{-1}$) during enzymatic treatment with 3.5 μg mL$^{-1}$ of laccase from *Trametes versicolor* (L*Tv*) at pH 7 and 40 °C.

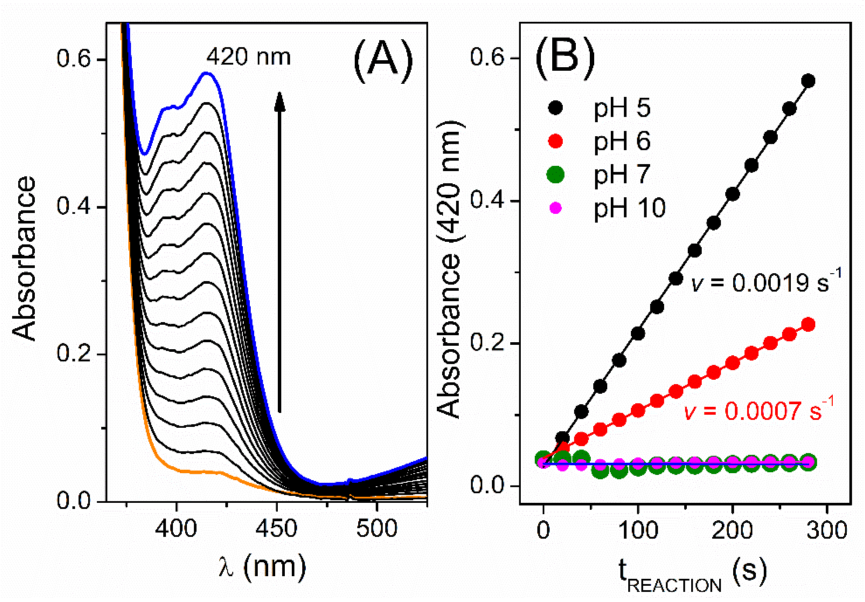


**Figure S3. (A)** Time-dependent evolution of the UV–Visible spectrum of ABTS during L*Tv*-catalyzed oxidation at pH 5. Spectra were recorded every 20 s from 0 to 300 s. **(B)** Variation in absorbance at 420 nm as a function of time during L*Tv*-catalyzed ABTS oxidation at pH 5, 6, 7, and 10. The reactions were conducted at 30 °C using 0.7 μg

$mL^{-1}$ of total soluble protein in the L*Tv* preparation and 9 x$10^{-5}$ M of ABTS.

**Table S2.** Comparison of the present combined photochemical-enzymatic treatment with previous studies on BTB removal from aqueous solution.

| Study | Method | Conditions (summary) | Reported removal | Ref. |
|---|---|---|---|---|
| Agarwal et al. (2016) | Adsorption using polyvinyl alcohol (PVA) as an adsorbent | Aqueous suspension of pH 6; contact time of 10 min; dose of 0.05 g of PVA; concentrations of 10-30 mg/L; T = 28-48°C. | Removal efficiency of 98.65%; maximum adsorption capacity of 276.2 mg/g according to the Langmuir model. | [15] |
| Ibrahim & Al-Hossainy (2020) | Chemical oxidation using the permanganate ion ($KMnO_4$) in acidic medium | Use of perchloric acid ($HClO_4$); constant ionic strength of 1.0 mol/dm³; T = 20-50 °C. | Efficient oxidation; pseudo-first order kinetic constants are reported (e.g., $k_{obs}$=0.58×$10^{-3}$$s^{-1}$ at 35 °C and acidic pH). | [16] |
| Soukeur et al. (2023) | Heterogeneous photocatalysis under sunlight using $MgFe_2O_4$ | Aqueous suspension of pH 3; catalyst dosage of 1 g/L; initial concentration of 10 mg/L; exposure to sunlight for 180 min. | 65% removal of the dye after 180 min of exposure; follows a first-order kinetic model. | [14] |
| Abbas (2021) | Direct photolysis under UV irradiation (without catalyst) for an analogous dye (bromophenol blue) | Irradiation at 254 nm; initial concentration of 10 mg/L; pH range 1-12 (optimum at pH 12); T = 20 °C. | Total disappearance of the dye after 150 minutes of irradiation. | [21] |
| This work - photochemical only | Direct UVC photolysis | pH 10, air/argon, 25 µg $mL^{-1}$ BTB. | $k_{PHOT}$ = 0.063-0.065 $min^{-1}$ (kinetics only; see Table S1 and Figure 3). | This study |
| This work - enzymatic only | Laccase (*T. versicolor*) | pH 5, 40 °C, Laccase 3.5 µg $mL^{-1,}$ 25 µg $mL^{-1}$ BTB. | $k_{ENZ}$ = 0.044 $min^{-1}$ (kinetics only; see Figure 5). | This study |
| This work - combined | Laccase → UVC photolysis (Sequence 2) | pH 5, 40 °C then UVC/air, 25 µg $mL^{-1}$ BTB, 140 min total. | 78% degradation; lowest ecotoxicity | This study |

| Study | Method | Conditions (summary) | Reported removal | Ref. |
|---|---|---|---|---|
| sequential (best case) | | | (~1% INH) among all treatments. | |

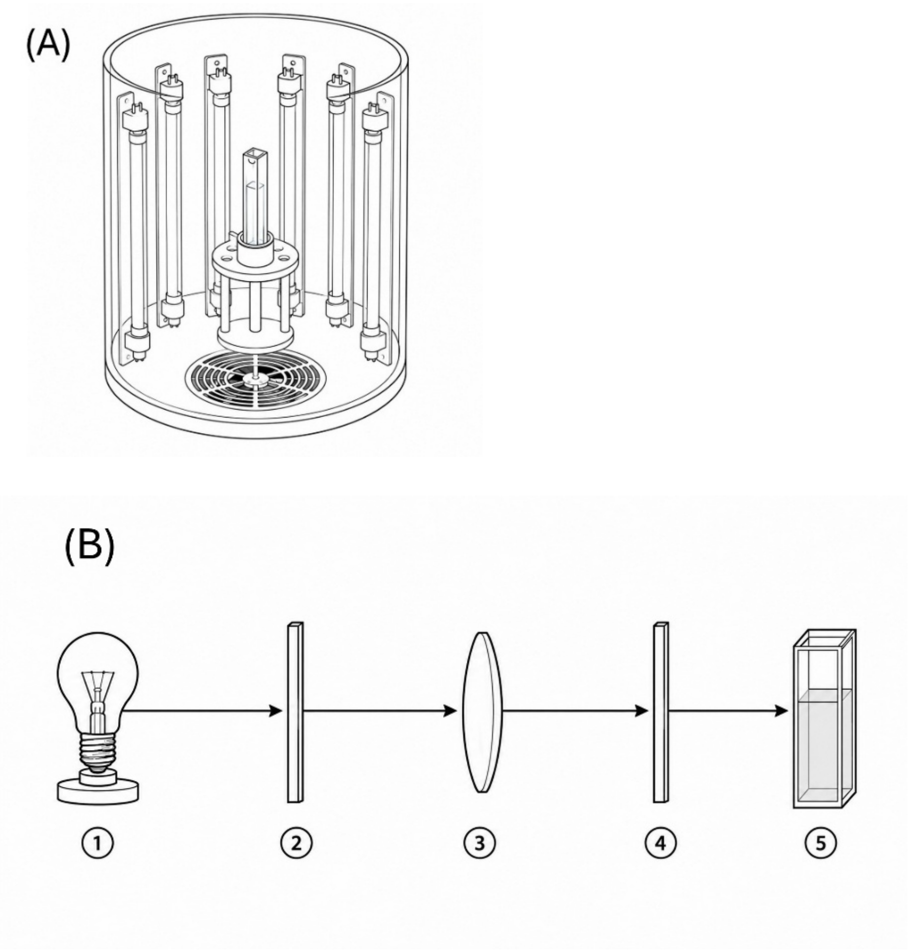


**Figure S4. (A)** Rayonet photochemical reactor (Rayonet Ltd., Branford, USA) used for UVC (254 nm), UVB (300 nm), and UVA (364 nm) irradiation, with three lamps per condition and irradiances of 4800, 4700, and 2600 µW/cm², respectively, at the surface of the quartz cell positioned at the center of the reactor. **(B)** Schematic representation of the home-made visible-light photoreactor: (1) 150 W quartz-halogen lamp, (2) UV cut-off filter, (3) converging lens, (4) thermal filter for infrared radiation removal, and (5) sample holder.
